# Understanding Adolescent Mental Health in the COVID-19 Era: A Psychodynamic Approach

**DOI:** 10.3390/children11070772

**Published:** 2024-06-26

**Authors:** Aslı Akın, Lea Sarrar

**Affiliations:** 1 Department of Psychology, Faculty of Sciences, MSB Medical School Berlin, 14197 Berlin, Germany; 2 Department of Psychiatry and Psychotherapy, Charité-Universitätsmedizin Berlin, 10117 Berlin, Germany; asli.akin@charite.de

**Keywords:** COVID-19 pandemic, adolescence, mental health, personality structure, psychodynamic conflicts, defense styles

## Abstract

Objective: This cross-sectional, case-control study aimed to explore the psychodynamic characteristics that influenced adolescents’ mental health during the COVID-19 pandemic. Methods: Personality structure impairments, psychodynamic conflicts, defense styles, and mental health issues were examined using the OPD-Structure- and Conflict-Questionnaires, the Defense Style Questionnaire, and the Patient Health Questionnaire in adolescents before (*n* = 288) and after (*n* = 451) the COVID-19 pandemic in Germany. Results: Adolescents with mental health issues exhibited greater impairments in personality structure, more immature defense styles, and higher levels of psychodynamic conflicts both before and after the pandemic onset. Comparisons between pre-pandemic and pandemic samples indicated a lower level of the conflict of taking care of oneself versus being cared for in passive mode among adolescents during the pandemic. In-depth analysis of adolescents with mental health issues from the pandemic group revealed significant associations between personality structure impairments and a maladaptive defense style with somatoform, depressive, anxiety, eating, and alcohol use disorders. Conclusion: The findings provide clinically relevant insights into the psychodynamic factors that contributed to the psychological vulnerability of adolescents during the COVID-19 pandemic. These insights can guide the development of targeted psychodynamic interventions to support adolescents’ mental health in similar future crises.

## 1. Introduction

Over the three-year span of the coronavirus disease 2019 (COVID-19) pandemic, the physical and mental health of the global population was at risk [1]. Alongside severe cases of infection and mortality, there was a marked increase in the prevalence of mental health issues following the pandemic’s onset [2,3]. Of particular concern was the impact on adolescents, who faced unique challenges during this period of uncertainty and upheaval [4,5,6]. Adolescents, already navigating a sensitive period of biological, social, cognitive, and emotional transition [7], were further affected by the imposition of social and educational restrictions, as well as ethical responsibilities and challenges, including quarantine of contacts, lockdowns, and vaccinations [8,9].

This global crisis challenged the mental health resilience of adolescents, revealing crucial insights into the psychological factors that contribute to the onset of mental health issues. Understanding these factors requires a comprehensive theoretical approach that can account for the complexities of adolescent psychology. Psychodynamic theory provides a clinically and empirically established framework for exploring these psychological factors that increase psychological vulnerability in adolescents, focusing on three core concepts: impairments in personality structure, psychodynamic conflicts, and defense mechanisms [10].

Personality structure, which mainly develops during the earliest attachment and relationship experiences, includes basic mental functions that regulate the self and its relationships with others [11]. With its sub-domains of attachment, identity, interpersonality, and control, the personality structure can achieve varying levels of structural functioning depending on the extent of impairments [10]. Low levels of structural functioning are linked to general psychopathology [12], making adolescents more susceptible to mental health issues during stressful times like a pandemic. Adolescents with impairments in personality structure struggle more with emotional regulation and interpersonal relationships [11], which could exacerbate the psychological impact of the pandemic.

Psychodynamic conflicts are defined as unconscious, temporally persistent, opposing motives, desires, values, and ideas within a person [13]. They arise from developmental tasks every child goes through and can concern the following conflictual needs and motives: closeness versus distance, submission versus control, taking care of oneself versus being cared for, self-worth conflict, guilt conflict, oedipal conflict, and identity conflict [10]. Psychodynamic conflicts shape experience and actions through either an active or passive mode of coping with them; while the passive mode involves unfulfilled desires like the need for security, the active mode involves defenses against these deficits, such as striving for self-sufficiency and suppressing attachment wishes [10]. According to the fundamental psychoanalytic theory, repressed psychodynamic conflicts that originate in childhood can be triggered by stressful events later in life and lead to the formation of symptoms [14], making adolescents more prone to anxiety, depression, and other mental health problems during the pandemic crisis.

Defense mechanisms are unconscious psychological strategies that help ward off unpleasant feelings [10,15]. These mechanisms become active when psychodynamic conflicts or structural impairments trigger too much emotional tension, preventing these feelings from becoming conscious [16]. While adaptive defense mechanisms ensure healthy and functional ways of dealing with increased emotional tension, neurotic and particularly maladaptive defense mechanisms are associated with psychopathology [15,17]. Adolescents who rely on maladaptive defense mechanisms may have experienced heightened psychological distress during the pandemic.

Overall, structural functioning, psychodynamic conflicts, and defense mechanisms represent psychological vulnerability factors that mainly develop throughout childhood and have a decisive influence on the ability to deal with stressful situations in adolescence and later adulthood [10]. The COVID-19 pandemic was a particularly stressful situation, or even a “global trauma” [18], that challenged people’s structural functioning, triggered psychodynamic conflicts, and required the use of defense mechanisms. Studies on adults have already confirmed the importance of psychodynamic characteristics for mental health during the pandemic, showing that defense mechanisms mediate the relationship between personality traits and post-traumatic symptoms [19] and predict adherence to COVID-19-related conspiracy theories [20]. A recently published study also demonstrated the positive relationship between impairments in personality functioning and psychological distress in adults during the pandemic [21].

Despite these findings, the specific psychodynamic vulnerability factors of adolescents during the pandemic remain underexplored. This study aims to elucidate the relevance of impairments in personality structure, psychodynamic conflicts, and defense mechanisms for the mental health status of adolescents during the COVID-19 pandemic. Specifically, we examined the psychodynamic characteristics of adolescents from clinical and non-clinical samples before and after the outbreak of the pandemic in Germany. In addition to a descriptive comparison of psychodynamic characteristics between adolescents with and without mental health issues, as well as before and during the pandemic, we investigated whether and to what extent various forms of mental health issues in adolescents during the COVID-19 pandemic can be explained by their psychodynamic characteristics. In sum, this approach aims to deepen our understanding of the complex psychodynamic interactions that shape adolescents’ mental health in times of crisis, enabling the development of targeted interventions to strengthen their resilience.

## 2. Materials and Methods

### 2.1. Sample

A total of 739 adolescents, aged between 14 and 21 years (*M*_age_ = 17.6; *SD* = 2.1; 61% female, 38% male, and 1% non-binary) took part in the study. Participants were recruited from outpatient clinics and educational institutions across Germany. Of the total sample, 39% (*n* = 288, *M*_age_ = 17.2; *SD* = 1.8; 64% female, 35% male, and 1% non-binary) were recruited and assessed before the outbreak of the COVID-19 pandemic in Germany (between March 2019 and February 2020). The remaining 61% (*n* = 451, *M*_age_ = 17.8; *SD* = 2.2; 58% female, 40% male, and 2% non-binary) were surveyed during the pandemic period (between March 2020 and March 2023). Among the 288 adolescents surveyed before the outbreak of the pandemic, 28% (*n* = 81) met the criteria for at least one mental disorder syndrome according to the Patient Health Questionnaire (PHQ-D) [22], while 72% (*n* = 207) showed no signs of mental health issues. Conversely, of the 451 adolescents surveyed during the pandemic, 43% (*n* = 192) met the criteria for at least one mental disorder syndrome according to the PHQ-D [22], while 57% (*n* = 255) showed no signs of mental health issues. Most participants were from high (41% of the pre-pandemic and 50% of the pandemic sample) or medium (39% of the pre-pandemic and 27% of the pandemic sample) socioeconomic backgrounds. Less than one third of the participants lived under low or very low socioeconomic conditions (20% of the pre-pandemic and 23% of the pandemic sample).

### 2.2. Measures

All participants were assigned to fill out a set of self-assessment questionnaires to measure their psychodynamic characteristics and mental health status. The questionnaires were administered by mail as paper-pencil versions. In order to prevent context effects, the questionnaires were given in a randomized order.

The OPD-CA2 Structure Questionnaire (OPD-CA2-SQ) [23] was used to assess impairments in personality structure. The questionnaire includes 81 items that assess impairments across four domains of personality structure, using a five-point scale ranging from no (0) to yes (4). Higher scores signify greater impairment in personality structure, indicating lower levels of structural functioning. The overall mean score of all items provides a general measure of personality structure impairments. For our analysis, we computed mean scores based on the raw scale scores, differing from the original authors’ scoring guidelines. The overall scale demonstrated very high reliability (McDonald’s ω = 0.97), and the sub-domains of attachment (McDonald’s ω = 0.86), identity (McDonald’s ω = 0.99), interpersonality (McDonald’s ω = 0.82), and control (McDonald’s ω = 0.90) showed high-to-very-high reliabilities in our sample. The results align with the similarly high internal consistencies reported in other studies [24].

The OPD-CA Conflict Questionnaire (OPD-CA-CQ) [25] was employed to assess the active and passive modes of coping with the seven psychodynamic conflicts. The questionnaire consists of 28 items, rated on a five-point scale from no (0) to yes (4). Higher average scores on the conflict scales indicate more pronounced psychodynamic conflicts. In this sample, the two-item scales turned out to be very reliable for the conflict of closeness versus distance in active (Spearman–Brown ρ = 0.97) and passive mode (Spearman–Brown ρ = 0.96), submission versus control in active (Spearman–Brown ρ = 0.98) and passive mode (Spearman–Brown ρ = 0.96), taking care of oneself versus being cared for in active (Spearman–Brown ρ = 0.98) and passive mode (Spearman–Brown ρ = 0.95), self-worth conflict in active (Spearman–Brown ρ = 0.98) and passive mode (Spearman–Brown ρ = 0.97), guilt conflict in active (Spearman–Brown ρ = 0.99) and passive mode (Spearman–Brown ρ = 0.97), oedipal conflict in active (Spearman–Brown ρ = 0.98) and passive mode (Spearman–Brown ρ = 0.96), and identity conflict in active (Spearman–Brown ρ = 0.97) and passive mode (Spearman–Brown ρ = 0.98). Prior psychometric investigations of the OPD-CA-CQ revealed inconsistent reliabilities for some subscales [26].

The Defense Style Questionnaire for Adolescents (DSQ-40-A) [27] was used to assess adaptive, neurotic, and maladaptive defense styles. This questionnaire comprises 40 items, representing 20 defense mechanisms, each with two items rated on a nine-point scale from not true (0) to completely true (8). Scores for defense mechanisms and defense styles are calculated by averaging the ratings for the relevant items, with higher scores indicating higher severity of the defense mechanisms or styles. The adaptive (McDonald’s ω = 0.54) and neurotic (McDonald’s ω = 0.50) defense style scales were sufficiently reliable, while the maladaptive defense style scale (McDonald’s ω = 0.75) demonstrated acceptable reliability in our sample. Previous psychometric evaluations of the DSQ-40 in adolescents have shown similar internal consistencies [28].

The PHQ-D [22] was utilized to screen for common mental disorders at the syndrome level. The 58-item questionnaire measures 16 conditions across five categories: somatoform, depressive, anxiety, eating, and alcohol use disorders. In this study, the somatoform syndromes scale (McDonald’s ω = 0.69) and depressive syndromes scale (McDonald’s ω = 0.80) showed acceptable-to-good reliabilities. Previous psychometric analyses of the PHQ-D have indicated excellent internal consistencies [29]. For the remaining diagnostic scales, calculating internal consistencies is deemed unnecessary, as these are primarily evaluated categorically with specific jump rules.

### 2.3. Data Analyses

Statistical analyses were conducted using IBM SPSS Statistics (Version 29). All tests were two-tailed, with a *p*-value < 0.05 considered statistically significant. Descriptive statistics, including means and standard deviations, were computed for all psychodynamic variables across each study group.

To compare impairments in personality structure, the use of adaptive, neurotic, and maladaptive defense mechanisms, and the extents of psychodynamic conflicts between adolescents with and without mental health issues as well as between the pre-pandemic and pandemic samples, separate univariate analyses of covariance (ANCOVA) were performed, controlling for sociodemographic variables (age, sex, and socioeconomic conditions). To control the type 1 error rate in multiple comparisons, the Bonferroni–Holm method was applied to all significance values. Partial η^2^ values were interpreted as follows: ≥0.01 as a small effect, ≥0.06 as a moderate effect, and ≥0.14 as a large effect [30].

To investigate the importance of psychodynamic characteristics for different types of mental health issues in adolescents during the COVID-19 pandemic, a series of binary logistic regression analyses were performed. Initially, the dependent variable was the general presence of mental health issues (coded as 0 for presence and 1 for absence of any mental health issues). Subsequently, syndromes from the PHQ-D [22] relating to somatoform, depressive, anxiety, eating, and alcohol use disorders were considered as dichotomous outcome variables (coded as 0 for presence and 1 for absence of the syndrome). The offered predictors were impairments in the overall and sub-domains of personality structure, the seven psychodynamic conflicts, and the three defense styles, as well as age, sex (coded as 0 for female and 1 for male sex), and socioeconomic conditions. Due to the exploratory nature of this study, independent variables were introduced into the regression models using a forward stepwise method, i.e., variables with *p* ≥ 0.05 were eliminated stepwise. Nagelkerke *R^2^* values were obtained for all models to estimate their explanatory power, with ≥0.20 interpreted as a small effect, ≥0.40 as a moderate effect, and ≥0.50 as a large effect [31].

## 3. Results

### 3.1. Comparisons between Adolescents with and without Mental Health Issues

Descriptive statistics, including means, standard deviations, and group comparisons for impairments in personality structure, defense styles, and psychodynamic conflicts among adolescents with and without mental health issues from the pre-pandemic and pandemic samples, are presented in Table 1.

### 3.2. Comparisons between Pre-Pandemic and Pandemic Samples

After adjusting for age, sex, and socioeconomic conditions, adolescents with mental health issues from the pre-pandemic and pandemic samples showed significant differences regarding the conflict of taking care of oneself versus being cared for in passive mode, *F*(1, 228) = 18.66, *p_adj_* = 0.003, partial η^2^ = 0.08. After adjusting for age, sex, and socioeconomic conditions, adolescents without mental health issues from the pre-pandemic and pandemic samples also showed significant differences regarding the conflict of taking care of oneself versus being cared for in passive mode, *F*(1, 404) = 3.93, *p_adj_* = 0.048, partial η^2^ = 0.01.

### 3.3. Predictors of Mental Health Issues during the COVID-19 Pandemic

Multiple binary logistic regression models were used to identify the psychodynamic predictors of mental health issues among adolescents during the COVID-19 pandemic. The results are summarized in Table 2.

The model predicting the general presence of mental health issues was statistically significant, χ^2^(3) = 111.35, *p* < 0.001, and explained a small amount of variance, Nagelkerke’s *R*^2^ = 0.35. Key predictors included impairments in overall personality structure, impairments in the personality structure domain of identity, and age.

The model predicting the presence of a somatoform syndrome was statistically significant, χ^2^(2) = 68.18, *p* < 0.001, explaining a small amount of variance, Nagelkerke’s *R*^2^ = 0.31. Impairments in the overall personality structure and age were significant predictors.

The model predicting the presence of a major depressive syndrome was statistically significant, χ^2^(2) = 128.61, *p* < 0.001, and explained a large amount of variance, Nagelkerke’s *R*^2^ = 0.51. Significant predictors included maladaptive defense style and impairments in overall personality structure.

The model predicting the presence of another depressive syndrome with impairments in the personality structure domain of interpersonality as the best predictor was statistically significant, χ^2^(1) = 5.00, *p* = 0.025, explaining a small amount of variance, Nagelkerke’s *R*^2^ = 0.04.

The model predicting the presence of a panic syndrome with impairments in the personality structure domain of attachment as the best predictor was statistically significant, χ^2^(2) = 39.84, *p* < 0.001, explaining a small amount of variance, Nagelkerke’s *R*^2^ = 0.30.

The model predicting the presence of another anxiety syndrome was statistically significant, χ^2^(3) = 67.48, *p* < 0.001, explaining a moderate amount of variance, Nagelkerke’s *R*^2^ = 0.46. Significant predictors included impairments in the personality structure domains of control and attachment, as well as age.

The model predicting the presence of an eating disorder syndrome with impairments in the personality structure domain of identity as the best predictor was statistically significant, χ^2^(1) = 18.89, *p* < 0.001, explaining a small amount of variance, Nagelkerke’s *R*^2^ = 0.17.

The model predicting the presence of harmful alcohol consumption was statistically significant, χ^2^(3) = 26.42, *p* < 0.001, explaining a small amount of variance, Nagelkerke’s *R*^2^ = 0.10. Significant predictors included maladaptive defense style, sex, and age.

## 4. Discussion

The present study aimed to explore the relevance of adolescents’ psychodynamic characteristics in relation to their mental health during the COVID-19 pandemic.

First of all, by comparing the mental health status of adolescents before and after the outbreak of the COVID-19 pandemic, evidence for the increased prevalence of mental health problems in adolescents after the outbreak of the pandemic could be provided. According to the observed cross-sectional data, the percentage of adolescents experiencing mental health issues increased from 28% to 43%, i.e., by a total of 15% after the outbreak of the pandemic. The discovered prevalence of mental health issues during the COVID-19 pandemic aligns with other studies [32,33,34], supporting the generalizability of the study results discussed below.

In accordance with psychodynamic theory, the findings of the study confirmed that adolescents with mental health issues exhibited higher impairments in personality structure, more frequent use of maladaptive and neurotic defense mechanisms, and higher extents of psychodynamic conflicts compared to their peers without mental health issues, both before and after the outbreak of the pandemic. In particular, the large mean differences between the clinical and non-clinical subgroups with regard to impairments in the overall and sub-domains of personality structure illustrate the relevance of the basic mental functions developed in the earliest years of life for general mental health [10,11]. There were also large mean differences between adolescents with and without mental health issues in terms of maladaptive defense style. Since defense mechanisms are understood as part of the personality structure and as a qualitative criterion for assessing it [10], this further underscores the high impact of psychodynamic characteristics developed early in life on mental health. In summary, impairments in personality structure and the use of maladaptive defense mechanisms appear to be general risk factors for the development of mental health issues in adolescents.

After applying the Bonferroni–Holm correction, we found almost no significant differences between adolescents from the pre-pandemic and pandemic samples regarding the psychodynamic characteristics. Interestingly, the only significant difference was found for the conflict of taking care of oneself versus being cared for in passive mode. This conflict was significantly higher in both adolescents with and without mental health issues before the pandemic, so it can be assumed that the outbreak of the pandemic decreased the extent of the conflict of taking care of oneself versus being cared for in passive mode. The central motive of this conflict is dependence in relationships and the need to receive attention, security, and care [10]. In the passive mode of coping with this conflict, demanding and clinging relationship behavior occurs and the fear of not being adequately cared for is constantly present [10]. Against the background of the consequences of the COVID-19 lockdown, such as stay-at-home-orders, school closures, and working from home, which led parents and their children to be closer together, it can be assumed that adolescents experienced increased care and attention in the parental home and thus could partially resolve this conflict. Adolescents, who are particularly confronted with the desire for independence and the simultaneous need for connection as part of the process of natural separation from their parents, may have experienced in the context of the COVID-19 pandemic that, if insecurity and danger arise in the outside world, they can find security and care in the parental home. The potential for such beneficial effects of the pandemic on family coexistence has already been demonstrated in a qualitative study by Subhadra et al. [35].

In turn, the fact that the extent of the impairments in personality structure and the defense styles hardly differ between the pre-pandemic and pandemic samples can be understood in light of the profound psychoanalytic theory of personality development. The psychoanalytic theory suggests that structural functioning and defense mechanisms are temporally and situationally stable unconscious aspects of personality—or rather *neurotic* dispositions [36]. However, these stable aspects of personality can become problematic and lead to mental illness when an individual faces particularly stressful life events.

Ultimately, the binary logistic regression analyses provided further insights into the associations between psychodynamic characteristics and various mental health syndromes in adolescents during the pandemic.

The regression models for predicting the presence of general and specific mental health issues indicated that impairments in personality structure and the use of maladaptive defense mechanisms are associated with a broad range of psychopathology during the pandemic. Accordingly, previous empirical studies that demonstrated the importance of personality structure and defense mechanisms for the mental health of adults during the COVID-19 pandemic could now be confirmed in adolescents [19,20,21].

In addition, the findings indicated that especially impairments in the personality structure sub-domain of identity appear to be a relevant risk factor for the development of mental health issues in adolescents during the pandemic. Khazand et al. [37] have already reported that the pandemic-related changes in adolescents’ social interaction opportunities—e.g., through closures of schools, distance learning, or the cancellation of free time activities—made the processes of exploring social roles and selecting peer groups, which are crucial for identity development, more difficult. The link between impairments in the sub-domain of identity and syndromes of eating disorders is in turn consistent with the tendency of adolescents, described by Klotter [38], to process identity problems through their bodies when other opportunities to experience self-determination are unavailable.

Another key finding of the regression analysis was the positive predictive effect of a maladaptive defense style on syndromes of major depression, eating disorders, and harmful alcohol consumption among adolescents during the pandemic. Maladaptive defense mechanisms, such as denial, splitting, and projection, which refuse the acceptance of reality and lead to a view of things as either all-good or all-bad [15], appear to have made it particularly difficult for adolescents to deal with the pandemic and contributed to various mental health problems. By additionally taking into account the significantly increased extent of the adaptive defense style in adolescents without mental health issues, the current investigation demonstrated the importance of defense mechanisms for the mental health state of adolescents during the COVID-19 pandemic.

Moreover, the regression analysis results revealed that panic and other anxiety syndromes in adolescents during the COVID-19 pandemic are primarily linked to impairments in the personality structure domain of attachment. This finding aligns with previous studies, which have demonstrated that insecure attachment styles predict COVID-19-related anxiety symptoms [39] and that attachment security has helped adolescents manage psychological stress and influenced their prosocial and health-protective behaviors during the pandemic [40].

In addition to the predictive relevance of psychodynamic characteristics for various syndromes of mental health issues, gender- and age-specific associations could be discovered.

Adolescents’ sex was negatively related to the presence of a somatoform syndrome and positively related to harmful alcohol consumption. Because of the small number of participants, adolescents with non-binary genders were excluded from the regression analyses, and female and male sex were considered as dichotomous variables. Accordingly, the results imply that female adolescents were more affected by a somatoform syndrome and male adolescents by harmful alcohol consumption during the COVID-19 pandemic. Previous studies have also pointed out the importance of gender-specific examinations of mental health problems during the pandemic [41,42].

Adolescents’ age was positively associated with the presence of general mental health issues, other anxiety syndromes, and harmful alcohol consumption, indicating that, the older adolescents were during the COVID-19 pandemic, the higher their risk of mental health problems was. This finding is in line with previous studies, which reported higher levels of mental health symptoms in older adolescents than in younger during the pandemic [43,44], but also with the discovery that older adolescents are at increased risk of mental disorders outside of the context of the pandemic [45].

Ultimately, it can be said that the present study had notable strengths, including a large sample of adolescents and control groups from the pre-pandemic period and adolescents without mental health issues, but also limitations which should be taken into account when interpreting the results. An important limitation is the cross-sectional design, which hinders causal interpretations of the relationships between psychodynamic characteristics developed early in life and the onset of mental health issues during the COVID-19 pandemic. Moreover, the study sample, while diverse, may not be fully representative of the broader adolescent population. The majority of participants were from medium to high socioeconomic backgrounds, which could limit the generalizability of the findings to adolescents from lower socioeconomic conditions. Additionally, it is important to consider the unequal distribution of adolescents with mental health issues within the subsamples, as well as the lack of consideration for clinically existing comorbidities. Furthermore, the use of self-report questionnaires may introduce bias due to subjective interpretation and response tendencies [46]. The method of participant recruitment through outpatient clinical facilities and educational institutions may have also introduced selection bias, as it might not capture adolescents who did not seek mental health support or were not enrolled in educational institutions during the pandemic. Future studies should employ longitudinal designs and incorporate multi-method approaches to provide a more comprehensive understanding of the psychodynamic factors influencing youth mental health during and beyond the pandemic.

## 5. Conclusions

The present cross-sectional, case-control study provides empirical evidence highlighting the importance of psychodynamic characteristics for the mental health status of adolescents during the COVID-19 pandemic. The findings demonstrate that syndromes of somatoform, depressive, anxiety, eating, and alcohol use disorders in adolescents during the pandemic were linked to impairments in overall and sub-domains of personality structure, as well as to a maladaptive defense style. Psychodynamic-oriented preventive and therapeutic interventions targeting these specific vulnerabilities might be effective in mitigating the impact of the pandemic on youth mental health. However, the conclusions should be interpreted with caution given the study’s limitations. Future research exploring these psychodynamic characteristics through longitudinal designs and incorporating a more diverse sample is needed to enhance our understanding and treatment of youth mental health in the context of unprecedented societal stressors.

## Figures and Tables

**Table 1:** Psychodynamic characteristics of adolescents with and without mental health issues before and during the COVID-19 pandemic.

	Pre-Pandemic Sample (*n* = 288)				Pandemic Sample (*n* = 451)			
	Adolescents with Mental Health Issues (*n* = 81)	Adolescents without Mental Health Issues (*n* = 207)	Comparison between Groups ^a^		Adolescents with Mental Health Issues (*n* = 192)	Adolescents without Mental Health Issues (*n* = 255)	Comparison between Groups ^a^	
Variable	*M* (*SD*)	*M* (*SD*)	*F*(1, 266)	η^2^	*M* (*SD*)	*M* (*SD*)	*F*(1, 366)	η^2^
Overall PS	1.65 (0.54)	1.08 (0.48)	72.59 *	0.22	1.75 (0.70)	1.04 (0.57)	94.19 *	0.21
Attachment	1.55 (0.63)	1.01 (0.50)	55.81 *	0.17	1.70 (0.73)	1.00 (0.54)	91.38 *	0.20
Identity	1.73 (0.59)	1.17 (0.55)	56.69 *	0.18	1.79 (0.76)	1.15 (0.55)	67.37 *	0.16
Interpersonality	1.62 (0.54)	1.07 (0.52)	59.75 *	0.18	1.71 (0.71)	1.03 (0.67)	77.58 *	0.18
Control	1.66 (0.64)	1.03 (0.59)	62.34 *	0.19	1.78 (0.82)	0.93 (1.00)	56.88 *	0.14
Adaptive DS	4.45 (1.23)	4.99 (1.13)	11.33 *	0.04	4.62 (1.38)	4.83 (1.34)	2.13	0.01
Neurotic DS	3.88 (1.05)	3.55 (1.05)	5.83 *	0.02	4.01 (1.13)	3.44 (1.33)	16.84 *	0.04
Maladaptive DS	3.17 (1.09)	2.37 (0.86)	46.81 *	0.15	3.21 (1.04)	2.37 (1.03)	59.61 *	0.14
C1a	1.23 (0.81)	0.93 (0.85)	6.57 *	0.02	0.98 (0.86)	0.26 (6.51)	1.05	0.00
C1p	1.73 (0.95)	1.45 (0.94)	3.57	0.01	1.71 (1.10)	1.01 (6.58)	0.66	0.00
C2a	1.16 (0.72)	1.01 (0.65)	2.18	0.01	1.06 (0.77)	0.60 (6.52)	0.48	0.00
C2p	1.72 (0.75)	1.46 (0.78)	6.98 *	0.03	1.83 (0.82)	1.03 (6.56)	1.22	0.00
C3a	1.05 (0.85)	0.80 (0.73)	6.72 *	0.03	1.32 (0.86)	0.44 (6.52)	1.62	0.00
C3p	1.86 (0.70)	1.91 (0.65)	0.84	0.00	1.34 (0.84)	1.02 (6.57)	0.19	0.00
C4a	1.61 (115)	1.39 (1.01)	2.10	0.01	1.75 (1.17)	1.05 (6.60)	0.87	0.00
C4p	1.48 (0.99)	0.91 (0.84)	21.33 *	0.07	1.53 (1.14)	0.56 (6.54)	1.78	0.01
C5a	0.29 (0.56)	0.13 (0.38)	8.58 *	0.03	0.55 (0.88)	−0.19 (6.46)	1.13	0.00
C5p	1.60 (1.00)	1.46 (0.98)	0.44	0.00	1.76 (1.10)	1.06 (6.59)	0.94	0.00
C6a	0.86 (0.94)	0.67 (0.87)	4.28 *	0.02	0.91 (0.97)	0.38 (6.54)	0.54	0.00
C6p	1.33 (0.86)	1.14 (0.88)	3.04	0.01	1.18 (0.99)	0.71 (6.55)	0.53	0.01
C7a	1.15 (0.86)	0.80 (0.72)	11.50 *	0.04	1.08 (0.98)	0.43 (6.53)	1.09	0.00
C7p	1.44 (1.16)	0.74 (0.89)	28.31 *	0.10	1.42 (1.24)	0.43 (6.54)	1.87	0.01

*Note. N* = 739. PS = personality structure. DS = defense style. C1a = conflict of closeness versus distance in active mode. C1p = conflict of closeness versus distance in passive mode. C2a = conflict of submission versus control in active mode. C2p = conflict of submission versus control in passive mode. C3a = conflict of taking care of oneself versus being cared for in active mode. C3p = conflict of taking care of oneself versus being cared for in passive mode. C4a = conflict of self-worth in active mode. C4p = conflict of self-worth in passive mode. C5a = guilt conflict in active mode. C5p = guilt conflict in passive mode. C6a = oedipal conflict in active mode. C6p = oedipal conflict in passive mode. C7a = identity conflict in active mode. C7p = identity conflict in passive mode. *M* = mean. *SD* = standard deviation. *F* = test value. η^2^ = partial eta squared. ^a^ Age, sex, and socioeconomic conditions were controlled * Bonferroni–Holm adjusted *p* ≤ 0.050.

**Table 2:** Binary logistic regression models predicting the presence of mental health issues in adolescents during the COVID-19 pandemic based on psychodynamic and demographic characteristics.

Variable	*B*	*SE_B_*	OR	95% CI	*p*
Model for predicting general mental health issues (*n* = 192)					
impairments in the overall personality structure	2.92	0.58	0.05	0.02, 0.17	<0.001
impairments in the personality structure domain of identity	1.13	0.52	3.09	1.12, 8.55	0.030
age	0.24	0.06	0.79	0.70, 0.88	<0.001
Model for predicting somatoform syndrome (*n* = 58)					
impairments in the overall personality structure	1.40	0.26	4.03	2.43, 6.69	<0.001
sex	−1.80	0.55	0.17	0.06, 0.49	0.001
Model for predicting major depressive syndrome (*n* = 66)					
impairments in the overall personality structure	2.55	0.41	12.76	5.71, 28.53	<0.001
maladaptive defense style	0.50	0.23	1.64	1.06, 2.55	0.028
Model for predicting other depressive syndrome (*n* = 31)					
impairments in the personality structure domain of interpersonality	0.63	0.28	1.88	1.08, 3.26	0.025
Model for predicting panic syndrome (*n* = 28)					
impairments in the personality structure domain of attachment	1.48	0.35	4.40	2.23, 8.67	<0.001
Model for predicting other anxiety syndrome (*n* = 31)					
impairments in the personality structure domain of control	1.69	0.55	5.40	1.83, 15.95	0.002
impairments in the personality structure domain of attachment	1.15	0.57	3.17	1.05, 9.60	0.041
age	0.32	0.14	1.38	1.05, 1.81	0.022
Model for predicting syndrome of eating disorder (*n* = 19)					
impairments in the personality structure domain of identity	1.48	0.35	4.38	2.19, 8.76	<0.001
Model for predicting harmful alcohol consumption (*n* = 131)					
maladaptive defense style	0.39	0.11	1.48	1.18, 1.84	<0.001
sex	0.60	0.24	1.83	1.14, 2.94	0.012
age	0.13	0.06	1.13	1.02, 1.27	0.026

*Note. N* = 192. *B* = regression coefficient. *SE_B_* = standard error of B. OR = odds ratio. CI = confidence interval.

## Data Availability

The datasets generated and analyzed during the current study are not publicly available due to confidentiality reasons. Requests to access the datasets should be addressed to L.S.

## References

[1] Onyeaka H., Anumudu C.K., Al-Sharify Z.T., Egele-Godswill E., Mbaegbu P. (2021). COVID-19 pandemic: A review of the global lockdown and its far-reaching effects. Sci. Prog..

[2] Nochaiwong S., Ruengorn C., Thavorn K., Hutton B., Awiphan R., Phosuya C., Ruanta Y., Wongpakaran N., Wongpakaran T. (2021). Global prevalence of mental health issues among the general population during the coronavirus disease-2019 pandemic: A systematic review and meta-analysis. Sci. Rep..

[3] Clemente-Suárez V.J., Martínez-González M.B., Benitez-Agudelo J.C., Navarro-Jiménez E., Beltran-Velasco A.I., Ruisoto P., Diaz Arroyo E., Laborde-Cárdenas C.C., Tornero-Aguilera J.F. (2021). The Impact of the COVID-19 Pandemic on Mental Disorders. A Critical Review. Int. J. Environ. Res. Public Health.

[4] Jones E.A.K., Mitra A.K., Bhuiyan A.R. (2021). Impact of COVID-19 on Mental Health in Adolescents: A Systematic Review. Int. J. Environ. Res. Public Health.

[5] Branje S. (2023). The impact of the COVID-19 pandemic on adolescent mental health across the world. Curr. Opin. Psychol..

[6] Meherali S., Punjani N., Louie-Poon S., Abdul Rahim K., Das J.K., Salam R.A., Lassi Z.S. (2021). Mental health of children and adolescents amidst COVID-19 and past pandemics: A rapid systematic review. Int. J. Environ. Res. Public Health.

[7] Kroger J. (2004). Identity in Adolescence.

[8] Longobardi C., Morese R., Fabris M.A. (2020). COVID-19 emergency: Social distancing and social exclusion as risks for suicide ideation and attempts in adolescents. Front. Psychol..

[9] Marrone M., Luca B.P.D., Stellacci A., Buongiorno L., Caricato P., Cazzato G., Ferorelli D., Solarino B., Stefanizzi P., Tafuri S. (2022). COVID-19 Vaccination in Italian Children: The Limits of Parental Rights. Children.

[10] Task Force OPD-CA-2 (2020). OPD-CA-2 Operationalisierte Psychodynamische Diagnostik im Kindes- und Jugendalter: Grundlagen und Manual [OPD-CA-2 Operationalized Psychodynamic Diagnosis in Childhood and Adolescence: Theoretical Basis and User Manual].

[11] Resch F., Parzer P. (2021). Psychic Structure, personality and psychodynamics. Adolescent Risk Behavior and Self-Regulation: A Cybernetic Perspective.

[12] Bach B., Simonsen S. (2021). How does level of personality functioning inform clinical management and treatment? Implications for ICD-11 classification of personality disorder severity. Curr. Opin. Psychiatry.

[13] Simmonds J., Constantinides P., Perry J.C., Drapeau M., Sheptycki A.R. (2015). Assessing psychodynamic conflict. Psychodyn. Psychiatry.

[14] Butler S.F., Demmin H., Strupp H.H., Heiden L.A., Hersen M. (1995). Psychodynamic psychotherapy. Introduction to Clinical Psychology.

[15] Cramer P. (2015). Defense mechanisms: 40 years of empirical research. J. Personal. Assess..

[16] Heinemann E., Hopf H. (2021). Psychische Störungen in Kindheit und Jugend [Mental Disorders in Childhood and Adolescence].

[17] Strandholm T., Kiviruusu O., Karlsson L., Miettunen J., Marttunen M. (2016). Defense Mechanisms in Adolescence as Predictors of Adult Personality Disorders. J. Nerv. Ment. Dis..

[18] Viscuso D.G.I., Mangiapane D.E. (2020). Pandemic COVID-19: Psychodynamic analysis of a global trauma. Clinical considerations pre\post Lock down. J. Med. Res. Health Sci..

[19] Gori A., Topino E., Palazzeschi L., Di Fabio A. (2021). Which personality traits can mitigate the impact of the pandemic? Assessment of the relationship between personality traits and traumatic events in the COVID-19 pandemic as mediated by defense mechanisms. PLoS ONE.

[20] Gioia F., Imperato C., Boursier V., Franceschini C., Schimmenti A., Musetti A. (2023). The role of defense styles and psychopathological symptoms on adherence to conspiracy theories during the COVID-19 pandemic. Sci. Rep..

[21] Kampe L., Hörz-Sagstetter S., Bohn J., Remmers C. (2024). How personality functioning relates to psychological distress and behavioral attitudes during the COVID-19 pandemic. Eur. Arch. Psychiatry Clin. Neurosci..

[22] Löwe B., Spitzer R.L., Zipfel S., Herzog W. (2002). Gesundheitsfragebogen für Patienten (PHQ-D). Manual und Testunterlagen [Patient Health Questionnaire (PHQ-D). Manual and Test Documents].

[23] Goth K., Schrobildgen C., Schmeck K. (2018). Das Inventar OPD-KJ2-SF (Operationalisierte Psychodynamische Diagnostik im Kindes- und Jugendalter—Strukturfragebogen). Deutschsprachige Version: Ein Fragebogen zur Selbstbeantwortung für die Erfassung der Persönlichkeitsstruktur im Jugendalter—Kurzbeschreibung. [The Inventory OPD-CA2-SQ (Operationalized Psychodynamic Diagnostics in Childhood and Adolescence—Structural Questionnaire). German Version: A Questionnaire for Self-Response for the Assessment of Personality Structure in Adolescence—Short Description]. Academic-Tests. https://academic-tests.com.

[24] Schrobildgen C., Goth K., Weissensteiner R., Lazari O., Schmeck K. (2019). Der OPD-KJ2-SF—Ein Instrument zur Erfassung der Achse Struktur der OPD-KJ-2 bei Jugendlichen im Selbsturteil [The OPD-CA2-SQ—An instrument for assessing the structure axis of the OPD-CA-2 in adolescents’ self-assessment]. Z. Kinder- Jugendpsychiatrie Psychother..

[25] Seiffge-Krenke I., Escher F.J. (2021). Entwicklung eines Fragebogens zur Selbst- und Fremdeinschätzung von OPD-KJ-Konflikten durch Patient_innen und Therapeut_innen [Psychodynamic conflicts: Development of a questionnaire for the assessment by patients and their therapists]. Z. Kinder- Jugendpsychiatrie Psychother..

[26] Cropp C., Claaßen B. (2021). Reliabilität und Validität des OPD-KJ-Konfliktfragebogens bei stationär behandelten Jugendlichen [Reliability and validity of the OPD-CA conflict questionnaire in an adolescent inpatient sample]. Z. Kinder- Jugendpsychiatrie Psychother..

[27] Schauenburg H., Willenborg V., Sammet I., Ehrenthal J.C. (2007). Self-reported defence mechanisms as an outcome measure in psychotherapy: A study on the German version of the Defence Style Questionnaire DSQ 40. Psychol. Psychother..

[28] Sarrar L., Goth K. (2022). Defense mechanisms reloaded in the light of impaired personality functioning: An attempt of clarification and simplification resulting in the DSQ-22-A for adolescents. Front. Psychiatry.

[29] Gräfe K., Zipfel S., Herzog W., Löwe B. (2004). Screening psychischer Störungen mit dem “Gesundheitsfragebogen für Patienten (PHQ-D)” [Screening for mental disorders using the “Patient Health Questionnaire (PHQ-D)”]. Diagnostica.

[30] Cohen J. (1988). Statistical Power Analysis for the Behavioral Sciences.

[31] Backhaus K., Erichson B., Plinke W., Weiber R. (2006). Multivariate Analysemethoden: Eine Anwendungsorientierte Einführung [Multivariate Analysis Methods: An Application-Oriented Introduction].

[32] Deng J., Zhou F., Hou W., Heybati K., Lohit S., Abbas U., Silver Z., Wong C.Y., Chang O., Huang E. (2023). Prevalence of mental health symptoms in children and adolescents during the COVID-19 pandemic: A meta-analysis. Ann. N. Y. Acad. Sci..

[33] Octavius G.S., Silviani F.R., Lesmandjaja A., Angelina, Juliansen A. (2020). Impact of COVID-19 on adolescents’ mental health: A systematic review. Middle East. Curr. Psychiatry.

[34] Nearchou F., Flinn C., Niland R., Subramaniam S.S., Hennessy E. (2020). Exploring the Impact of COVID-19 on Mental Health Outcomes in Children and Adolescents: A Systematic Review. Int. J. Environ. Res. Public Health.

[35] Evans S., Mikocka-Walus A., Klas A., Karantzas G., Westrupp E.M. (2020). From “it has stopped our lives” to “spending more time together has strengthened bonds”: The varied experiences of Australian families during COVID-19. Front. Psychol..

[36] O’Reilly J., Gibbons R., O’Reilly J. (2021). Psychodynamic Theory: The development of a model of the mind. Seminars in the Psychotherapies.

[37] Khazand S., Riley T.N., Whitener M.P., Zapolski T.C.B. (2021). Who am I: Considerations for adolescent development during a pandemic. J. Ment. Health Soc. Behav..

[38] Klotter C. (2016). Essstörungen und Identität [Eating disorders and identity]. Identitätsbildung über Essen.

[39] Vismara L., Lucarelli L., Sechi C. (2022). Attachment style and mental health during the later stages of COVID-19 pandemic: The mediation role of loneliness and COVID-19 anxiety. BMC Psychol..

[40] Coulombe B.R., Yates T.M. (2022). Attachment security predicts adolescents’ prosocial and health protective responses to the COVID-19 pandemic. Child. Dev..

[41] Dal Santo T., Sun Y., Wu Y., He C., Wang Y., Jiang X., Li K., Bonardi O., Krishnan A., Boruff J.T. (2022). Systematic review of mental health symptom changes by sex or gender in early-COVID-19 compared to pre-pandemic. Sci. Rep..

[42] Prowse R., Sherratt F., Abizaid A., Gabrys R.L., Hellemans K.G., Patterson Z.R., McQuaid R.J. (2021). Coping with the COVID-19 pandemic: Examining gender differences in stress and mental health among university students. Front. Psychiatry.

[43] Duan L., Shao X., Wang Y., Huang Y., Miao J., Yang X., Zhu G. (2020). An investigation of mental health status of children and adolescents in China during the outbreak of COVID-19. J. Affect. Disord..

[44] Zhou S.-J., Zhang L.-G., Wang L.-L., Guo Z.-C., Wang J.-Q., Chen J.-C., Liu M., Chen X., Chen J.-X. (2020). Prevalence and socio-demographic correlates of psychological health problems in Chinese adolescents during the outbreak of COVID-19. Eur. Child. Adolesc. Psychiatry.

[45] Kessler R.C., Petukhova M., Sampson N.A., Zaslavsky A.M., Wittchen H.-U. (2012). Twelve-month and lifetime prevalence and lifetime morbid risk of anxiety and mood disorders in the United States. Int. J. Methods Psychiatr. Res..

[46] Demetriou C., Ozer B.U., Essau C.A., Cautin R.L., Lilienfeld S.O. (2015). Self-report questionnaires. The Encyclopedia of Clinical Psychology.

